# Early neurological development and nutritional status in Mexican socially deprived contexts

**DOI:** 10.1371/journal.pone.0270085

**Published:** 2022-06-21

**Authors:** Edson Serván-Mori, Evelyn Fuentes-Rivera, Amado D. Quezada, Carlos Pineda-Antunez, María del Carmen Hernández-Chávez, Angélica García-Martínez, Abby Madrigal, Raquel García-Feregrino, Tania Santiago-Angelino, María Hernández-Serrato, Lourdes Schnaas

**Affiliations:** 1 Center for Health Systems Research, National Institute of Public Health, Cuernavaca, Morelos, Mexico; 2 Center for Demographic, Urban and Environmental Studies, College of Mexico A.C, Mexico City, Mexico; 3 Center for Evaluation and Surveys Research, The National Institute of Public Health, Cuernavaca, Morelos, Mexico; 4 Department of Developmental Neurobiology, The National Institute of Perinatology Isidro, Espinosa de los Reyes, Mexico City, Mexico; 5 Lucy Family Institute for Data and Society, University of Notre Dame, Notre Dame, IN, United States of America; 6 Center for Information on Public Health Decisions, The National Institute of Public Health, Cuernavaca, Morelos, Mexico; Kaohsuing Medical University Hospital, TAIWAN

## Abstract

Early childhood development (ECD) is a critical stage in the intergenerational process of human development. Targeted interventions depend on accurate and up-to-date ECD measurements. This paper presents estimates for the nutritional and neurodevelopmental status of socially marginalized children in Mexico. We performed a cross-sectional study based on data collected in 2019–2020 during home visits to 1,176 children aged 0–38 months across 24 highly marginalized locations in Oaxaca. We assessed nutritional status according to the World Health Organization 2006 child-growth standards and ECD status using the Child Development Evaluation Test, 2^nd^ Edition. We stratified results by sex. Prevalence of stunting was 5.3 percentage points (p.p.) higher (*p* = 0.023) in males (25.3%; 95% CI: 20.2%, 31.1%) compared to females (20.0%; 95% CI: 15.0%, 26.1%). Overall prevalence rates stood at 5.7% (95% CI: 4.0%, 8.1%) for underweight, 1.5% (95% CI: 0.9%, 2.7%) for wasting and 3.6% (95% CI: 2.3%, 5.7%) for overweight/obesity, with no significant differences by sex. Prevalence of normal development was 8.3 p.p. lower (*p* = 0.001) in males (39.3%; 95% CI: 34.5%, 44.4%) compared to females (47.6%; 95% CI: 41.6%, 53.6%). By development area, the highest prevalence of suboptimal outcomes among children with developmental lag or at risk of delay was observed in their gross motor and language skills: 24.1% (95% CI: 20.0%, 28.8%) and 38.6% (95% CI: 34.0%, 43.3%), respectively. The largest difference between the sexes was found in the language area. Our results show that childhood development strategies have been insufficient thus far in the studied population. Programs specifically designed to prevent ECD lags and bridge inequality gaps are urgently needed.

**Trial registration:** ClinicalTrials.gov ID: NCT04210362.

## 1 Introduction

Early childhood development (ECD), a central axis in the formation of human capacities, plays a crucial role in the intergenerational process of human development [1]. It is essential, not only to wellbeing, but also to long-term development and economic growth. ECD represents a progressive, multidimensional, and comprehensive process that runs from gestation to the fifth year of life. When optimal, it translates into the construction of increasingly complex capacities that contribute to the development and full exercise of the rights of individuals.

ECD requires sensitive care, that is, the mindful concern of family members and caregivers for vital aspects of development such as health, nutrition, and safety. This includes paying perceptive attention to children and seizing early learning opportunities for their development. It is important to recognize that ECD benefits the most from an adequate environment during the first three years of life [2–4]. Suboptimal ECD not only affects children; it also undermines the society in which they develop [5]. Failure to intervene during the first years of life is associated with high health costs, wasted capabilities and low academic and labor productivity. This, in turn, deepens social inequalities [6]. Studies of low- and middle-income countries (LMICs) have shown that the cognitive abilities of girls and boys (hereafter children) as well as pre-adolescents are positively associated with income levels [7, 8]. Inequality among population groups is partially the result of the parent-child transmission of socioeconomic levels [9]. This mechanism impedes the accumulation of the human capital required to escape poverty [10].

The need to effectively support ECD was acknowledged in the International Development Agenda, where it is explicitly addressed in the first four Sustainable Development Goals (SDGs 1–4) [11]. The SDG call for action stresses the pressing need to design and implement ECD interventions, particularly for socially vulnerable populations.

While the socioeconomic lag among highly marginalized communities in Mexico [12] has been widely documented, little has been written about the ECD conditions of the children in those contexts [13, 14]. Data provided by national surveys such as the 2018 National Health and Nutrition Survey (ENSANUT, by its initials in Spanish) exclude detailed information on the specific areas of development. Similarly, the 2019 and 2020 waves of the survey have omitted information on neurodevelopment in children. This can be partially explained by the recent failure to allocate a specific budget to early childhood, thus limiting not only the availability of information on highly vulnerable population groups, but also the possibility of designing targeted, rational and sustainable interventions aligned with a comprehensive public policy on early childhood, as specified in the National Strategy for Integrated Early Childhood Development (ENAPI, by its initials in Spanish) [15]. The ENAPI is concerned with recovering the trajectories of children who risk falling short of their full potential and promoting the realization of their rights.

The dearth of information on ECD is also partially attributable to the methodological challenges posed by its multidimensional nature. Availability of accurate and up-to-date ECD measurements is essential, not only to ascertain the current status of childhood development, but also to appraise the impact of related interventions and understand how their results can be used to design and implement effective public policies [16]. Moreover, ECD measurements lay the foundation for the ENAPI itself as well as for other important lines of action.

Using information gathered in 2019 and part of 2020, we offer a descriptive analysis of recent and original data regarding two areas of interest intimately linked to ECD: nutritional status and neurodevelopment [17, 18]. We performed analyses by sex and compared prevalence between males and females. Our work focused on children 0–38 months old living in the socially marginalized municipalities of Oaxaca, one of the most economically and socially deprived states in Mexico [19–22].

## 2 Methods

### 2.1 Design/Participants

We conducted a cross-sectional study based on primary data collected from 1,179 children 0–38 months old in the state of Oaxaca, in Mexico. The 24 municipalities covered were characterized by high and very high levels of social marginalization [23]. All of them exceeded the national and most of them the state-level averages of poverty, educational lags and restricted access to health services, social security coverage, quality housing space and food [24]. The children analyzed had participated in a baseline measurement exercise performed during an impact assessment of the Neurological and Psycho-affective Development Program (PDNyP by its initials in Spanish). This Program was designed and implemented by the civil society organization Un Kilo de Ayuda, A.C. A detailed description of the assessment study has been published elsewhere [25].

In 2019 and 2020, we collected data concerning the anthropometric measurements, sociodemographics, health status, ECD and household characteristics of the selected children. To identify the children of interest and their primary caregivers, we established preliminary contact with the latter and inquired about their interest in participating in the study. For this first encounter, we used two mechanisms: (1) we extended an invitation to the municipal authorities, explaining the objectives of our project and requesting their support in organizing a meeting with the population of interest. During the meeting, we described the PDNyP and the activities in our research project; (2) we identified potential participants by reviewing the household censuses of the selected municipalities. To this end, the municipal authorities allowed us to consult their household registers. We then visited the homes of children aged three years or younger to explore the interest of the primary caregivers in participating in our study. Having identified the eligible children, we obtained sample measurements from July 2019 to March 2020 for 1,176 children aged 0–38 months.

### 2.2 Data collection

A questionnaire was administered during home visits to collect data on the study population, namely the characteristics of the children from 0–38 months old, their mothers or primary caregivers, the members of their households and their housing conditions. Data was also gathered on household assets and income. Anthropometric measurements of the children were taken and their ranking in childhood development scales was determined. The contents of the questionnaire have been detailed elsewhere [25].

We designed the questionnaires using the Research Electronic Data Capture (REDCap^TM^) web application for creating and managing online surveys and databases [26]. We then piloted and administered the instruments using mobile phones equipped with the Android^®^ Operative system. Qualified and standardized personnel performed the anthropometric measurements in conformity with international protocols [27, 28]. Trained, standardized and certified personnel with ample experience applied the child development test and processed the results. They also administered the instrument used to explore sociodemographic and housing features as well as the characteristics of the primary caregivers and other household members. The research team controlled the quality and replicability of the results according to data management protocols designed and tested by the team members.

Participants from the study population joined our project on a voluntary basis, having provided written informed consent and without being offered any form of economic compensation for their involvement. Our research protocol, questionnaires, measurement techniques and informed consent letters were approved by the Research, Ethics and Biosecurity Committees of the National Institute of Public Health, Mexico (1538/CI-896-2018/CB18-317) and by ClinicalTrials.gov (ID: NCT04210362).

### 2.3 Measurements

#### Nutritional status

We assessed the nutritional status of the children according to the World Health Organization (WHO) 2006 child-growth standards, on the basis of weight, height and age. Anthropometric measurements were taken according to the Lohman technique, using SECA 872^TM^ digital scales and SECA 206^TM^ portable stadiometers. Personnel standardized under the Habicht method [28] performed the measurements. Following WHO criteria [29, 30], underweight was defined as Z weight for age < -2, stunting as height-for-age Z < -2, overweight or obesity as body mass index for age Z > 2 and wasting as weight-for-height Z < -2. All observations were within the intervals for identifying valid observations: -6.0 to +5.0 for weight for age Z, -6.0 to +6.0 Z for length or height for age Z, -5.0 to +5.0 for BMI for age Z and weight for length or height Z.

#### Neurological development

We applied the Child Development Evaluation Test, 2^nd^ edition (EDI-II, by its acronym in Spanish) [31, 32], designed to detect developmental lags and risk of developmental delay across 14 age groups within the range of 1–60 months. The EDI-II assesses five development areas: gross motor, fine motor, language, social and cognitive skills. The first four sections of the test are applicable at all ages, while the cognitive skills section centers exclusively on children aged 37 months or older. Apart from measuring development areas, this test explores the general neurological status of children and the presence of biological risk factors related to the prenatal care of mothers as well as complications experienced by the newborns; it also addresses warning signs. Validated for Mexican children using the Batelle Developmental Inventory, 2^nd^ Edition, as gold standard, the EDI-II test offers 81% sensitivity (95% CI: 75%, 86%) and 61% specificity (95% CI: 54%, 67%) [33]. It requires directly observing the children and formulating questions for their primary caregivers. Results are interpreted according to a traffic-light scale where green denotes normal development, yellow developmental lag, and red risk of developmental delay. Except for a few age-related specifics, the general classification scheme of the EDI-II test can be summarized as follows: green indicates that all activities for the corresponding age group have been successfully performed; yellow that not all activities for the corresponding age group have been successfully performed but those for the preceding age group have; and red means that not all activities for the corresponding or preceding age group have been successfully performed. Overall assessment covers every development area, a neurological exploration, the presence of alarming or warning signs and the presence of biological risk factors. Children who obtain a red classification need to be referred to an appropriate specialist for a more thorough evaluation [31].

#### General and sociodemographic characteristics

Individual characteristics of the children included age (months), gestational age (weeks), birth weight (grams), preterm birth (birth prior to 37 weeks gestation), number of siblings and health-care coverage, if any. Characteristics of the primary caregiver included relationship to the child (mother, father, another relative or a non-relative), age category (<18, 18–19, 20–24, 25–29, 30 to 39 and ≥40 years), marital status (single, married/in union or widowed/divorced/separated), schooling (elementary or none, middle, and high school or beyond), employment status during the last week (worked or not). Household characteristics included family structure (a nuclear family consisting of a married couple with unmarried children or other types of family), indigenous status (households whose head, her/his spouse or ascendants declared that they spoke an indigenous language) [34], overcrowding (an average of more than two household members per room) [35], number of household members, enrollment in a government social program (yes or no), household materials and services (presence of a dirt floor, gas stove, type of access to running water, and possession of durable goods such as a motor vehicle, a phone line or mobile phone, and household appliances).

### 2.4 Data analysis

We described the general and sociodemographic characteristics of the study sample, their primary caregivers and households, using medians and interquartile intervals (IQI) for continuous variables, and frequencies and percentages for categorical variables. Prevalence rates for stunting, underweight, wasting and overweight/obesity, as well as for child development outcomes from the EDI-II test, were obtained through logit back-transformed 95% confidence intervals (CIs), with standard errors (SEs) adjusted for clustering of observations within municipalities [36]. To assess the association between the overall outcome of the EDI-II test and nutritional status indicators, we estimated polychoric correlations and their standard SEs. All analyses were performed overall and by sex. Differences in prevalence between the sexes were tested using Wald tests. We employed the Stata MP v. 17.0 statistical package for all analyses [37].

## 3 Results

Our analytical sample comprised a total of 1,142 children (n = 617 male and n = 525 female) with valid information on either the child development outcomes (n = 1,131 total, n = 610 male and n = 521 female) or nutritional status indicators (n = 1,134 total, n = 613 male, n = 521 female); n = 34 out of n = 1,176 children (2.8%) lacked information on some or all outcomes. A descriptive analysis of the general and sociodemographic characteristics of the analytical sample, their primary caregivers and their households is shown in Table 1. The median age of the children was 19.8 months (IQI: 12.5, 27.2); of these, 8.2% had been born preterm, and 77.5% were covered by a health-insurance service, with Seguro Popular being the most frequent (64.7%). In relation to the characteristics of the primary caregivers, 91.2% were the mothers, 8.2% were adolescents (aged 15–19 years), 84.1% were married or in a union, 39.6% had attended high school or enjoyed some level of higher education and 40.8% had worked during the week preceding the interview. Regarding household characteristics, 79.2% consisted of nuclear families (a married couple with unmarried children); 28.3% participated in a government social program; 29.5% were indigenous; and 21.8% lived in overcrowded homes. Regarding housing materials and services, approximately 10% of the households had dirt floors, 75% a gas stove and 54.4% running water inside the house. The general characteristics of the children, their primary caregivers and their households were evenly distributed between males and females.

**Table 1:** General and sociodemographic characteristics of the analytical sample by sex.

	Male	Female	Overall
	n = 617	n = 525	n = 1,142
Child characteristics			
Age, months	20.5 [12.7,27.3]	19.4 [12.4,26.6]	19.8 [12.5,27.2]
Gestational age, weeks	39.0 [38.0,40.0]	39.0 [38.0,40.0]	39.0 [38.0,40.0]
Preterm (gestational age <37 weeks)	49 (8.11%)	43 (8.29%)	92 (8.19%)
Birth weight, g	3150 [2800,3430]	3100 [2800,3400]	3140 [2800,3400]
Number of siblings			
None	212 (38.1%)	155 (32.8%)	367 (35.7%)
One	210 (37.8%)	185 (39.1%)	395 (38.4%)
Two or more	134 (24.1%)	133 (28.1%)	267 (25.9%)
Health insurance			
Seguro Popular	389 (63.0%)	350 (66.7%)	739 (64.7%)
Social Security	85 (13.8%)	50 (9.52%)	135 (11.8%)
Private or other	3 (0.486%)	5 (0.952%)	8 (0.701%)
Mixed	3 (0.486%)	0 (0%)	3 (0.263%)
None	137 (22.2%)	120 (22.9%)	257 (22.5%)
Caregiver characteristics			
Relationship with the child			
Mother	561 (90.9%)	480 (91.4%)	1041 (91.2%)
Father	8 (1.30%)	8 (1.52%)	16 (1.40%)
Other	48 (7.78%)	37 (7.05%)	85 (7.44%)
Age, years			
15 to 17	13 (2.11%)	9 (1.71%)	22 (1.93%)
18 to 19	37 (6.01%)	35 (6.67%)	72 (6.31%)
20 to 24	165 (26.8%)	124 (23.6%)	289 (25.3%)
25 to 29	167 (27.1%)	159 (30.3%)	326 (28.6%)
30 to 39	162 (26.3%)	143 (27.2%)	305 (26.7%)
40 or higher	72 (11.7%)	55 (10.5%)	127 (11.1%)
Marital status			
Single	61 (9.93%)	50 (9.58%)	111 (9.77%)
Married/in union	516 (84.0%)	439 (84.1%)	955 (84.1%)
Widowed/separated/divorced	37 (6.03%)	33 (6.32%)	70 (6.16%)
Schooling			
Elementary or none	118 (19.2%)	104 (19.9%)	222 (19.5%)
Middle	251 (40.8%)	214 (40.9%)	465 (40.9%)
Highschool or higher	246 (40.0%)	205 (39.2%)	451 (39.6%)
Worked during past week	249 (40.4%)	217 (41.3%)	466 (40.8%)
Household characteristics			
Nuclear home^1^	490 (79.4%)	414 (78.9%)	904 (79.2%)
Beneficiary of a government social program	163 (26.4%)	160 (30.5%)	323 (28.3%)
Indigenous household^2^	188 (30.5%)	149 (28.4%)	337 (29.5%)
Number of household members	4 [3, 5]	4 [4, 6]	4 [4, 5]
Overcrowding^3^	132 (21.7%)	113 (21.8%)	245 (21.8%)
House materials and services			
Dirt floor	52 (8.52%)	60 (11.6%)	112 (9.93%)
Gas stove	460 (75.5%)	391 (75.5%)	851 (75.5%)
Access to running water			
Inside the house	328 (53.9%)	285 (55.0%)	613 (54.4%)
In the terrain (outside the house)	155 (25.5%)	98 (18.9%)	253 (22.4%)
Other^4^	126 (20.7%)	135 (26.1%)	261 (23.2%)
Possession of durable goods			
Motor vehicle	195 (32.5%)	184 (35.6%)	379 (33.9%)
Phone line or mobile phone	496 (81.8%)	415 (80.3%)	911 (81.1%)
Household appliances	506 (83.5%)	435 (84.3%)	941 (83.9%)

Estimates are median [P25, P75] for continuous variables and frequencies (%) for categorical variables. In some cases, the sum of frequencies over all categories of a variable does not exactly match the total due to the presence of missing values. For binary indicator variables, only the indicating category is shown.

1Both father and mother of the child live in the household.

2Households whose head, her/his spouse or ascendants declared that they spoke an indigenous language

3More than two household members per room

4Public source, from a tanker trunk or carried from other place (e.g. well, river, lake)

**Fig 1:**
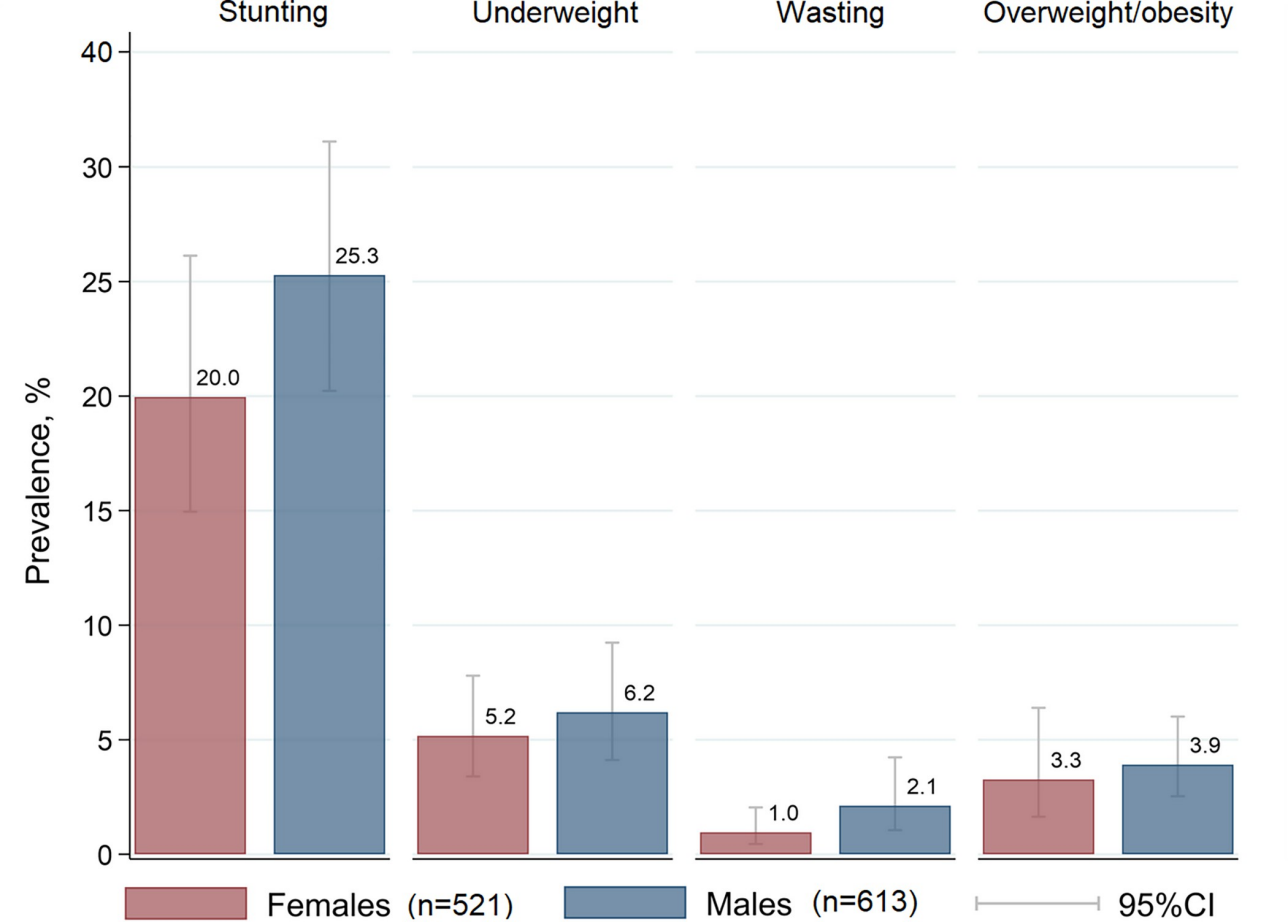
Nutritional status prevalence for male and female children.

Prevalence of stunting was 22.8% (95% CI: 18.2%, 28.2%) and 5.3 ± 2.2 (±SE) percentage points (p.p.) higher (*p* = 0.023) in males (25.3%; 95% CI: 20.2%, 31.1%) compared to females (20.0%; 95% CI: 15.0%, 26.1%) (Fig 1). The overall prevalence rates were 5.7% (95% CI: 4.0%, 8.1%) for underweight, 1.5% (95% CI: 0.9%, 2.7%) for wasting and 3.6% (95% CI: 2.3%, 5.7%) for overweight/obesity, with no significant differences by sex.

**Fig 2:**
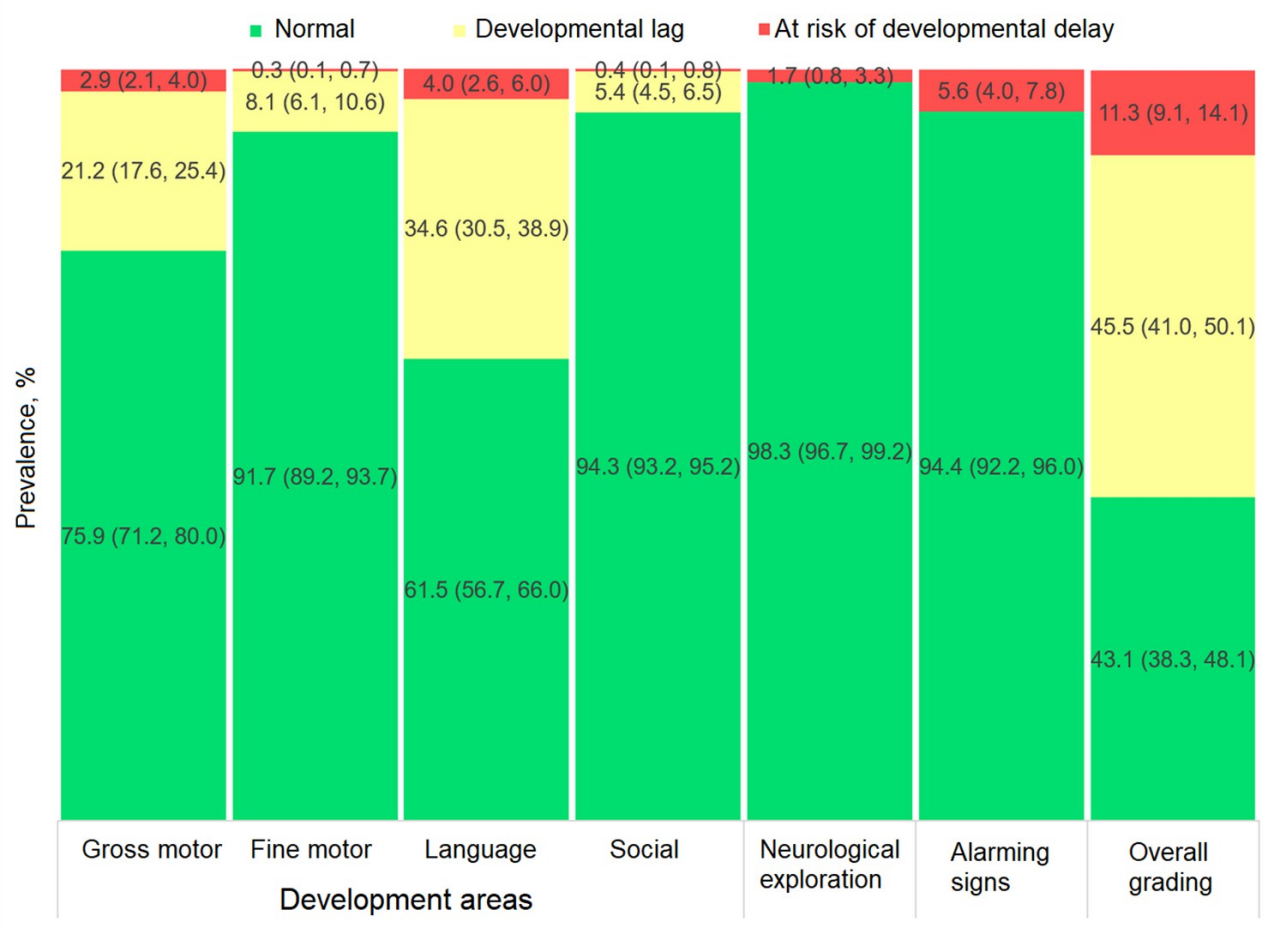
Prevalence of child development categories from the EDI-II test (n = 1,131).

**Fig 3:**
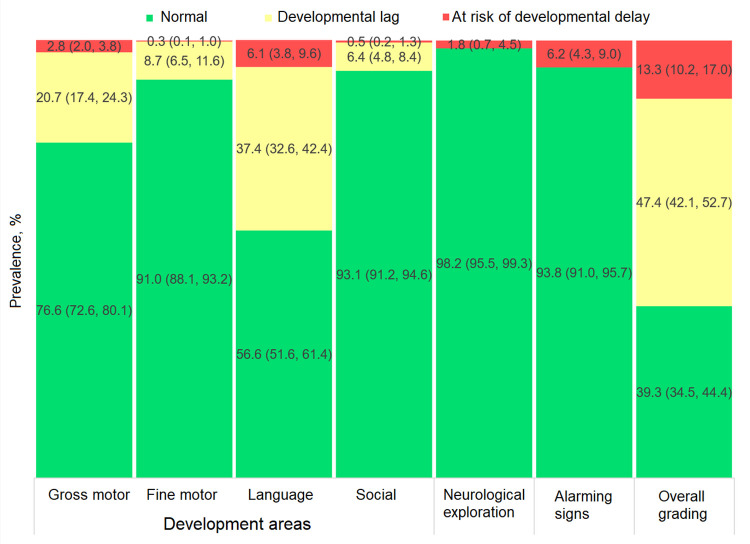
Prevalence of child development categories from the EDI-II test among males (n = 610).

**Fig 4:**
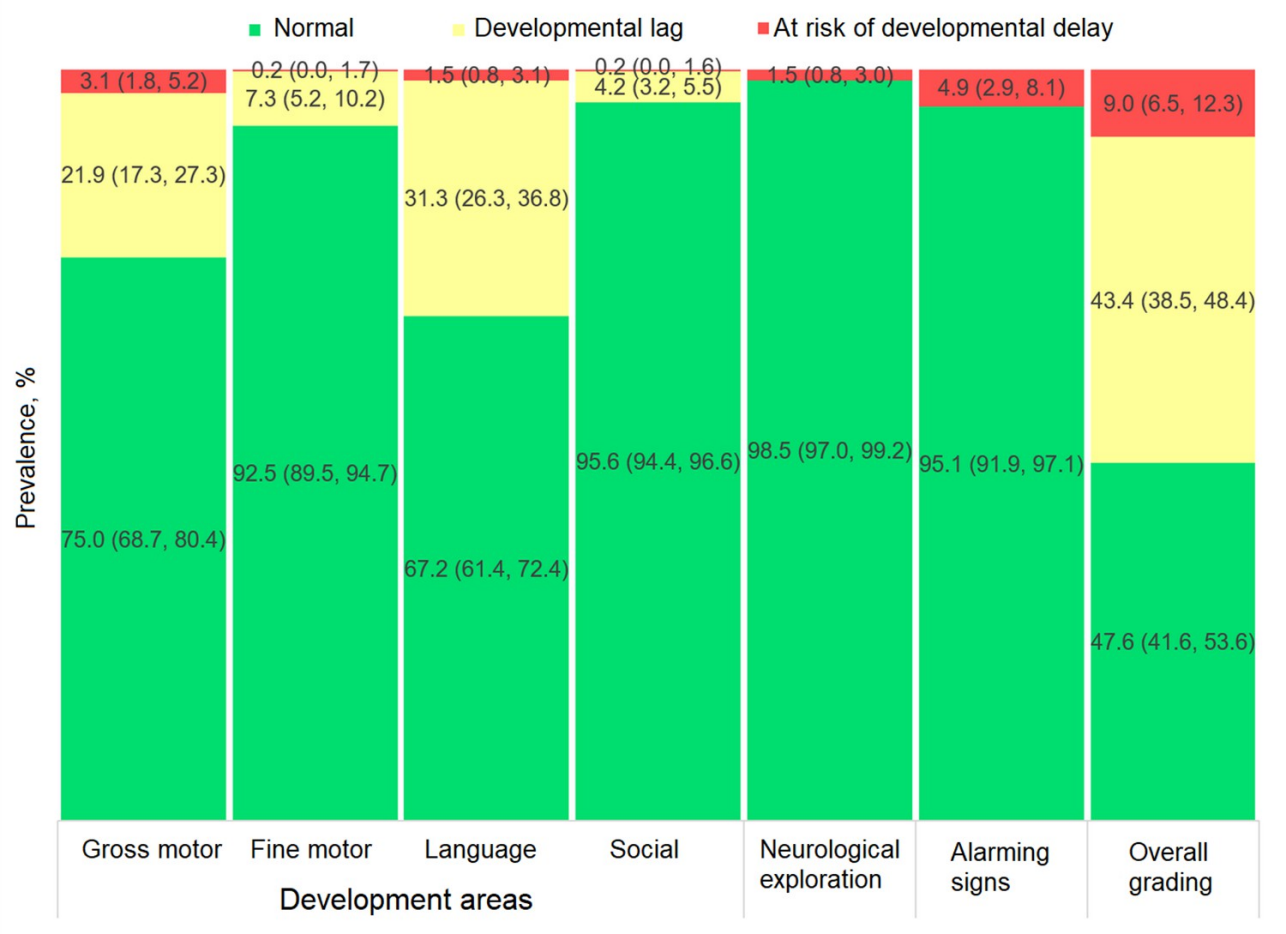
Prevalence of child development categories from the EDI-II test among females (n = 521).

As for the overall grading of ECD status, 43.1% of children (95% CI: 38.3%, 48.1%) exhibited normal development, 45.5% (95% CI: 41.0%, 50.1%) developmental lag and 11.3% (95% CI: 9.1%, 14.1%) risk of developmental delay (Fig 2). By development area, the highest prevalence of suboptimal outcomes among children with developmental lag or at risk of delay was observed in their gross motor and language skills: 24.1% (95% CI: 20.0%, 28.8%) and 38.5% (95% CI: 34.0%, 43.3%) respectively (adding these two development categories, Fig 2). Results by sex are shown in Figs 3 and 4, with normal development prevalence registering 8.3 ± 2.2 p.p. lower (*p* = 0.001) in males (39.3%; 95% CI: 34.5%, 44.4%) compared to females (47.6%; 95% CI: 41.6%, 53.6%). The largest difference between sexes was found in the language area, with males showing a prevalence of normal development 10.6 ± 2.1 p.p. lower (*p* < 0.001) than females.

Among males, the polychoric correlations between the overall child development outcome (coded from red to green) and the nutritional status indicators were –0.23 ± 0.06 for stunting, -0.34 ± 0.09 for underweight, -0.17 ± 0.13 for wasting and 0.04 ± 0.12 for overweight/obesity. For females, they were -0.14 ± 0.07 for stunting, -0.13 ± 0.11 for underweight, -0.35 ± 0.19 for wasting and 0.28 ± 0.11 for overweight/obesity. Overall, the correlations were –0.20 ± 0.05 for stunting, -0.25 ± 0.07 for underweight, -0.24 ± 0.11 for wasting and 0.14 ± 0.09 for overweight/obesity.

## 4 Discussion

This study described the ECD status of highly vulnerable children in Mexico. It set a precedent by providing information on gaps observed from the first years of life, aggravated by the lack of comprehensive ECD interventions in the context of sensitive care (Nurturing Care during ECD) [38], a strategy promoted by the WHO, the World Bank and UNICEF to foster human-capital development and social mobility as part of the 2030 SDGs [2]. Our results shed light on policies pertaining to childhood development that need to be strengthened if Mexico is to meet the 2030 SDGs. The SDG plan of action urgently calls upon countries to ensure that all children fully exercise their rights and attain effective access to health-care services during early childhood [11].

Besides the SDGs 2030 agenda Mexico, as all the countries in the world, must now face the unprecedent crisis that the COVID-19 pandemic and the war in Ukraine have brought. Specifically, the COVID-19 pandemic had impacted critical sectors needed to reduce ECD gaps. Problems as food insecurity and poor-quality diets, reduced income and limited financial resources, limited care and restricted health services, interrupted education for children and adults and, unhealthy household environment have been reinforced in marginalized communities due to the pandemic [39]. The war in Ukraine has also contribute with the dramatic inflation, to the worsening of these critical sectors to improve ECD performance in marginalized populations. These limitations imposed by the pandemic and the war could lead to an erosion of the already poor ECD levels mostly in the nutritional component unless urgent actions are taken to fight back the impacts [40–42].

Acknowledging the existence of vast social inequalities in ECD has been an essential step in the establishment of programs aimed at reducing development gaps. Such is the case of the Chile Grows with You (*Chile crece contigo*) Program, a comprehensive system launched by Chile in 2007 to protect children. This program arose from the identification of social inequalities, salient among them a 30% prevalence of developmental lags in children under five. The prevalence reported by Chile is lower than that observed in our study [43, 44].

One major challenge facing ECD in Mexico is the dearth of measurements for developmental components, rendering it difficult to identify developmental lags in children. The estimates of the 2018 ENSANUT revealed that only 13% of children between 12 and 59 months old had undergone ECD assessment and less than a fourth of children with developmental delay had ever been offered follow-up [45].

If ECD measurement efforts are not intensified, the needs of children with developmental lags will remain unmet. This problem is particularly severe in the most highly marginalized communities in Mexico. Those covered by our study faced a greater risk of developmental delay (11.3%) than similar communities in Puebla (6.2%) and Coahuila (4.3%) [46]. The prevalence we observed was also significantly higher than that reported by the 2018 ENSANUT, where the national average came to 2.8%; however, it should be noted that the ENSANUT used developmental scales not directly comparable with ours and included children under five years [47]. Likewise, prevalence of delay under the EDI yellow and red coding scheme in our study (56.8%) was greater than that identified in other Mexican communities (approximately 16%) [45, 48]. Although these figures were reported by studies not focused exclusively on highly marginalized communities–one of them conducted with PROSPERA Program participants–they bring to light the area of opportunity that disadvantaged populations offer for the implementation of targeted interventions. The magnitude of ECD prevalence identified in our study demonstrates that developmental delay is a public-health problem in Mexico and other countries. For example, the prevalence of developmental delay, as measured by the Ages and Stages instrument, has been reported to be as high as 40% in communities suffering from extreme poverty in China [49].

Highly marginalized communities in Mexico are dominated by negative contextual factors that translate into potential barriers to maintaining a favorable atmosphere for sensitive childcare. As a result, suboptimal ECD levels persist, with children finding themselves caught in the poverty trap. According to the National Institute of Statistics, Geography, and Information Technology (INEGI, by its initials in Spanish), 27.6% and 16.9% of households in highly marginalized communities such as Guerrero and Oaxaca, respectively, were overcrowded in 2017, as opposed to a national average of 9.4% [50]. Indigenous ethnicity is another axis of inequality in society and specifically in minors, depending on the type of service analyzed. According to 2012 estimates, 9.5% of all young children in Mexico live in households where an indigenous language is spoken [51]. Among the highly marginalized communities in our study, the figure virtually tripled that amount (29.5%).

The nutritional status of children plays a prominent role in ECD, with our polychoric correlation analysis confirming an association between malnutrition and low levels of overall child neurodevelopment. Our analysis of nutritional problems in the highly marginalized locations of Oaxaca yielded higher prevalence than those reported by the 2018 ENSANUT-100K [52]; for instance, according to our figures, underweight stood at 5.7% vs. 4.4% reported by the ENSANUT [52]. However, the largest gap occurred in stunting, where our estimates yielded a prevalence of 22.8%, compared to 14.9% reported by the ENSANUT-100K. Disaggregating the survey sample into tertiles of socioeconomic level, the prevalence of stunting in our study proved higher than that reported by the ENSANUT for the poorest tertile (17.5%) [52]. In 2018, the National Council for the Evaluation of Social Development Policy (CONEVAL, by its initials in Spanish) published data on regional differences regarding chronic malnutrition among children under five. Using data collected in 2015, their estimates showed a significantly lower prevalence in the northwest (8.5%) vs. the south, including the state of Oaxaca (16.7%) [53].

All the above suggest that ECD strategies need to consider more comprehensive and equitable designs that incorporate various dimensions of development. They must not only address education and child-rearing, but also integrate health care, food, water, hygiene and social services. In addition, ECD strategies should offer communities nutritional guidance based on locally available foods that are fresh, varied and nutritious, rather than relying exclusively on micronutrient supplementation. This will provide children with optimum nourishment, beginning with exclusive breastfeeding for the first six months of life, and the introduction of nutritionally adequate and safe solid foods at six months together with continued breastfeeding up to the age of two years or beyond. Diet must be accompanied by the conditions necessary for children to reach their full physical and intellectual potential [54].

Compared to females, male children in our study exhibited greater disadvantages in the gross motor and language areas of development. They obtained lower prevalence of normal development, and therefore higher prevalence of developmental lag or risk of delay. In regard to nutritional status, males were at greater disadvantage as well, showing higher prevalence of stunting by 5.3 p.p. Our results are in line with previous studies indicating that male children are more vulnerable than female children in highly marginalized contexts [55, 56].

Among the highly marginalized communities analyzed in Oaxaca, only 28% of households reported participating in a social program and only 12% enjoyed Social Security coverage. However, the low levels of ECD identified not only indicate inequitable access to services; they also point to a failure to respect the rights of children. According to the 1948 Universal Declaration of Human Rights, “everyone has the right to a standard of living adequate for the health and wellbeing of himself/herself and his/her family, including food, clothing, housing and medical care as well as necessary social services” [57]. Children in communities such as those in our study are deprived of their rights, as they live in deprived contexts. They lack adequate access to the initial endowment of skills required to face the barriers in their environments. Nearly half of the children in our sample suffered from developmental lags. As a result, these children will be deprived of the tools needed to lift themselves out of poverty, and will remain in the grip of intergenerational social vulnerability.

The prevalence of stunting, developmental lags and risk of developmental delay observed in our study sample clearly confirms the persistence of gaps in marginalized locations. Existing programs have failed to cover all the regions in the country. A variety of health programs in Mexico and other Latin American countries provide care for pregnant mothers and their offspring in early childhood; however, they do not communicate with one another, much less collaborate synergistically. Their dissociation is particularly evident in programs from other social sectors, with the result that interventions and services repeatedly overlap and at times operate at cross purposes. In this respect, and with the view of effectively addressing the persisting ECD lags in disadvantaged areas, the role of parents and other caregivers must be considered. Programs need to utilize the knowledge, skills, time and material resources of parents and caregivers as part of their interventions for the provision of adequate childcare. These assets can translate into parental education programs on child-rearing, attaching greater importance to active community participation, which goes further than merely receiving financial support. The development of local capacities is particularly relevant in those localities suffering from the sharpest social inequities. It is essential that sectoral education, health, and wellbeing programs include specific lines of action that place the interests of children at the center of their work. These efforts must evolve within a framework of affectionate and sensitive care emphasizing the promotion of health. According to the Lancet series published in 2017, this is part of effective intervention packages for pregnancy and early childhood [58, 59].

The results of our work can serve to reinforce existing ECD projects such as the ENAPI. This initiative promotes intersectoral participation among communities, educational agents, and health personnel. Although it has been approved and has developed guidelines for implementation at the three levels of government, the ENAPI has encountered a series of obstacles in its attempts to initiate operations. At the federal level, the executive branch has shown a lack of interest in allotting a budget for early childhood programs. At the state and municipal levels, the services and personnel of the SIPINNA need to be reinforced. Adequate capacities are required to evaluate ENAPI services and interventions for the comprehensive development of all Mexican children during early childhood [60].

Despite some advances in ECD, Mexico has failed to respond in a coordinated manner to share the responsibilities at the federal, state and local levels for scaling up and extending the services where they are urgently needed. As many as 72% of households in our study reported not participating in a social program. This situation is not likely to improve given the termination of programs such as PROSPERA [61], one of the most targeted maternal and childhood strategies in the country. PROSPERA was replaced with a scholarship program for families with school-aged children (Benito Juarez Scholarships for the Wellbeing). However, the latter does not include health or nutrition components, e.g., nutritional supplementation for children under two, self-care workshops and Well-Child Checkups, all important for childhood development.

Among the limitations of our study was the extraction of data only from participants who had access to our invitation. Nonetheless, we measured both participants who enrolled in the PDNyP and those who attended the PDNyP information sessions but declined to participate. Voluntary attendance may have led to the exclusion of families uninterested in or unaware of our informative sessions. Our results may have thus underestimated the number of caregivers who did not respond because of a lack of interest in the early development of their children. Conversely, they may have overestimated the number of caregivers who enrolled in other programs with overlapping or added services. Similarly, highly marginalized communities may have been underrepresented, as some of the remote localities in the state may have not had the opportunity to access our invitation. Nonetheless, the marginalized locations where we gathered information concentrated a population characterized by high levels of vulnerability in the state of Oaxaca. Our inferences are limited to the sampling frame of communities from our original study design as well as participants within communities who were able to attend the information sessions and agreed to participate in the present study, but not necessarily in the PDNyP project. Our results may provide valuable information on ECD and nutritional status for other communities in Mexico that share similar socioeconomic characteristics as those analyzed in our study.

In sum, our study found a high prevalence of suboptimal ECD in a sample of children with similar characteristics to those living in other marginalized communities in Mexico and examined the future implications of this condition for the health and development of children in general. From the viewpoint of the rights of children, the decision to invest fresh resources explicitly and forcefully in strategies that have been shown to favorably impact the prevention of ECD lags is clearly in the hands of public-policy makers. Only then will children be able to realize their full potential. This is crucial for achieving sustainable development, ending the intergenerational transmission of poverty, closing inequality gaps and promoting development in the Mexican population.
